# Allogeneic stem cell transplantation in acute leukemia patients after COVID-19 infection

**DOI:** 10.1038/s41409-021-01225-w

**Published:** 2021-02-09

**Authors:** Maximilian Christopeit, Mirjam Reichard, Christian Niederwieser, Radwan Massoud, Evgeny Klyuchnikov, Nicolas Haase, Christine Wolschke, Francis Ayuk, Silke Heidenreich, Nicolaus Kröger

**Affiliations:** Department of Stem Cell Transplantation, University Medical Center Hamburg-Eppendorf, Hamburg, Germany

**Keywords:** Infectious diseases, Leukaemia

## To the Editor

The risk for severe and fatal courses of COVID-19 is particularly high after allogeneic hematopoietic stem cell transplantation (alloSCT) [1–4]. Cardiovascular and renal morbidity is increased post COVID-19 [5]. Neither data nor recommendations exist regarding alloSCT in survivors of COVID-19 [6, 7]. Seven patients (*n* = 5 acute myeloid leukemia, AML, ELN intermediate or adverse risk, *n* = 2 acute lymphatic leukemia, ALL, each one standard risk with induction failure, BCRABL positive) with an indication for alloSCT who had survived COVID-19 were referred to our department. They had been diagnosed with AML/ALL between December 2019 and April 2020 and contracted COVID-19 in March/April 2020 during a hospital stay. COVID-19 had been diagnosed briefly after admission in 3 patients, 4 patients had received the diagnosis of COVID-19 2 weeks, 2 months, 2 months and 4 months after diagnosis of acute leukemia. Four patients had been infected during or briefly before induction chemotherapy/prephase chemotherapy. Three patients were infected during salvage therapy (Blinatumomab, FLAG-Ida).

COVID-19, measured as time from first PCR [8, 9] positive throat and nose swab (TNS) to first of consecutive negative TNS had lasted 45 (median, range 12–70) days. ICU treatment had been necessary for 6 patients for 9 (median, range 2–22) days, 3 patients had been mechanically ventilated for 4, 4, and 12 days. Specific treatment had included plasma of convalescent donors, Lopinavir/Ritonavir, and pentaglobin. ARDS, deep vein thrombosis, sepsis and catheter related blood stream infection had complicated COVID-19. Inflammation parameters were high. Anti-SARS-CoV-2 antibodies (Diasorin SARS-CoV-2 IgG (Diasorin, Italy) and Elecsys Anti-SARS-CoV-2 Ig assay (Roche, USA)) were found in 5/7 patients, 2 were antibody negative. The leukemia remission status necessitated antineoplastic treatment in all patients during the phase of COVID-19. COVID-19 courses of the patients are summarized in Table 1.

**Table 1:** Patients’ characteristics.

Median age (range) – years	60 (34–69)
Female/male	2/5
AML (adverse risk, intermediate risk, ELN 2017)	5 (3, 2)
ALL (BCRABL positive, standard risk with induction failure)	2 (1, 1)
Disease status at Tx	
AML CR/refractory	4/1
ALL CR_BCRABL+_/ALL CR_MRD-_	1/1
*COVID-19 period*	
COVID-19 severity: asymptomatic, mild illness, moderate illness, severe illness, critical illness	0, 0, 0, 4, 3
Duration of ICU stay (6 patients), median (range) days	9 (2–22)
Duration of mechanical ventilation (3 patients), days	4, 4, 12
Ferritin, median (range) µg/l, peak during COVID-19 phase	4003 (593–15,356)
IL-6, median (range) ng/l, peak during COVID-19 phase	538 (50–1513)
Procalcitonin, median (range) µg/l, peak during COVID-19 phase	5 (0–17)
CrP, median (range) mg/l, peak during COVID-19 phase	292 (185–368)
SARS-CoV-2 IgG, median (range) AU/ml during COVID-19 phase	43 (0–345), 2 patients without IgG
*Allogeneic stem cell transplantation period*	
SARS-CoV-2 IgG, median (range) AU/ml, at start of conditioning	109 (0–331), 2 patients without IgG
Time first negative SARS-CoV-2 TNS – alloSCT, median (range) days	94 (64–136)
Thoracic computed tomography	
No evidence of COVID-19 typical infiltrations	1
Evidence of COVID-19 typical infiltrations (remnants, decreasing)	6
Signs of etiologically different pneumonia (consistent with mold)	2
Pulmonary function testing	
Restrictive airway disease	2
TLC	in these patients 74%, 70%
VC	in these patients 67%, 75%
DLCOc, median (range)	63% (45–87%)
Obstructive airway disease	0
Conditioning MAC/RIC	6/1
TBI, Gray	4, 1x 8 Gy, 3x 12 Gy
GvHD prophylaxis	
ATG	3^a^
Post transplantation cyclophosphamide	5
Immunosuppression	
Tacrolimus, mycophenolic acid	4
Cyclosporin A, mycophenolic acid	3
Donor	
Haploidentical related	4
Matched related	1
Mismatched unrelated	2
Patient blood group	
A	3
B	0
AB	0
0	4
Donor blood group	
A	3
B	1
AB	0
0	3
Patient CMV IgG positive/negative	3/4
Donor CMV IgG positive/negative	4/3
Donor/patient CMV IgG matching	
Both negative	3
Both positive	3
Donor negative, patient positive	0
Donor positive, patient negative	1
CD34+ cells × 10^6^/kg bodyweight	7.2 (6.3–8.7)
Leukocyte engraftment, median (range) days	17 (14–31), all patients engrafted
Thrombocyte engraftment, median (range) days	23 (19–43), 2 patients without thrombocyte engraftment
Infectious complications (%)	
FUO	3
Pneumonia	2
CMV reactivation	1
Acute GvHD	4
Overall grade 1	2
Overall grade 2	2
Gastrointestinal tract	4
Skin	0
Polyserositis (pretibial edema, pericardial effusion, pleural effusion)	1
Follow up after alloSCT median in days (range)	77 (40–109)

*alloSCT* allogeneic stem cell transplantation, *AML* acute myeloid leukemia, *ALL* acute lymphoblastic leukemia, *ATG* antilymphocyteglobulin, *AU* arbitrary units, *CMV* cytomegalovirus, *DLCOc* corrected diffusing capacity of lung for carbon monoxide, *ELN* European Leukemia Net, *FUO* fever of unknown origin, *GvHD* graft versus host disease, *MRD* measurable residual disease, *TLC* total lung capacity, *TNS* throat-nose-swab, *VC* vital capacity.

^a^One patient received ATG and post transplant cyclophosphamide due to the presence of donor specific antibodies.

Institutionally and with health authorities, consent was developed to devise a dedicated team (7 physicians, 8 nurses, 1 physiotherapist) to treat these patients during the period of alloSCT in a designated area. Psycho-oncological care was provided via phone consultation. Patients were single-room-isolated. Separate rooms with air management (e.g., HEPA filtration) were not available. Staff was protected by specific single-use gear. Potential reactivation of SARS-CoV-2 was PCR-monitored by TNS (3×/week), oral throat rinses and plasma testing (each 1×/week). Patients were also regularly tested for SARS-CoV-2 IgM and IgG antibodies. Additionally, every patient received a thoracic CT scan in order to assess morphologic lung infiltrations after COVID-19. Beyond this, usual standard operating procedures were followed. Antimicrobial prophylaxis consisted of ciprofloxacin, acyclovir, cotrimoxazole or alternative anti-pneumocystis drug, micafungin or alternative antifungal, detailed elsewhere [10]. Written informed consent was adapted to the situation.

At the time of admission for alloSCT, all patients tested negative for SARS-CoV-2 (TNS). Thoracic computed tomography (CT) at admission revealed small remnants of former COVID-19 infiltrations in 6 patients. Signs of pneumonia other than COVID-19 were visible in 2 patients, consistent with pulmonary mold infection. One patient was without signs of pneumonia. Pulmonary function testing immediately before the start of conditioning revealed moderate airway restrictions in 2 patients, with total lung capacity of 74%/70% and vital capacity of 67%/75%. Corrected diffusing capacity of lung for carbon monoxide (DLCOc) was between 45% and 87%, median 63%. No obstructive lung disease was evident. Time from onset of COVID-19 to alloSCT was 133 (median, 106–171) days, time from resolution of COVID-19 (first negative TNS) to alloSCT was 94 (64–136) days. Disease characteristics were adverse with many primary induction failures and molecular failures. Conditioning was myeloablative for 6 patients, 4 including total body irradiation (3 × 12 Gray, 1 × 8 Gray). Patients’ characteristics are depicted in Table 1. After a median follow-up of 77 (range 40–109) days, no reactivation of SARS-CoV-2 occurred. Leukocyte engraftment was reached in all patients after 14–31 (median 17) days. The 52 patients consecutively transplanted with peripheral blood stem cells in the same institution from April to May 2020 showed leukocyte engraftment after 11 (median, range 7–21) days (*p* = 0.0003, Mann–Whitney test). Thus, though comparison is flawed by a high degree of mismatch transplantation in the 7 patients reported here, time to leukocyte engraftment is significantly longer. Thrombocyte engraftment ≥20.000/µl was reached in 5 patients after 19–43 days (median 23 days) with 2 patients still in need of prophylactic thrombocyte infusions. Acute graft versus host disease (aGvHD) developed in 4 patients, 2 of which were grade 2 of the gastrointestinal tract and 2 grade 1, each *n* = 1 gastrointestinal tract and polyserositis. Steroidrefractory aGvHD necessitated treatment with Ruxolitinib in 1 patient. Infectious complications included fever of unknown origin, pneumonia and CMV reactivation, and did not lead to an intensive care unit stay.

The patients (*n* = 5) who had developed antibodies against SARS-CoV-2 remained antibody positive after transplant. Two patients never showed antibodies against SARS-CoV-2. All patients were discharged alive. Four patients had to be readmitted due to complications (each *n* = 1 sinuisoidal obstruction syndrome, relapse, infection, alloimmune phenomena).

Taken together, alloSCT post severe COVID-19 is feasible even 1–3 months after resolution of infection. We did not observe reactivation of SARS-CoV-2. On the basis of the 7 patients presented, we tend to negate a correlation between a complicated course of COVID-19 before and a complicated course after transplantation. Larger analyses will have to evaluate if a history of SARS-Cov2 infection does present a significant risk for alloSCT with a complicated course.

## References

[1] Altuntas F, Ata N, Yigenoglu TN, Basci S, Dal MS, Korkmaz S, et al. COVID-19 in hematopoietic cell transplant recipients. Bone Marrow Transplant. 2020. 10.1038/s41409-020-01084-x. 10.1038/s41409-020-01084-x 33110186

[2] Malard F, Genthon A, Brissot E, van de Wyngaert Z, Marjanovic Z, Ikhlef S (2020). COVID-19 outcomes in patients with hematologic disease. Bone Marrow Transplant.

[3] Roedl K, Heidenreich S, Pfefferle S, Jarczak D, Urbanowicz TT, Nörz D (2021). Viral Dynamics of SARS-CoV-2 in Critically Ill Allogeneic Hematopoietic Stem Cell Transplant Recipients and Immunocompetent Patients with COVID-19. Am J Respir Crit Care Med.

[4] Aydillo T, Gonzalez-Reiche AS, Aslam S, van de Guchte A, Khan Z, Obla A (2020). Shedding of Viable SARS-CoV-2 after Immunosuppressive Therapy for Cancer. N Engl J Med.

[5] Puelles VG, Lutgehetmann M, Lindenmeyer MT, Sperhake JP, Wong MN, Allweiss L (2020). Multiorgan and Renal Tropism of SARS-CoV-2. N Engl J Med.

[6] Ljungman P, Mikulska M, de la Camara R, Basak GW, Chabannon C, Corbacioglu S (2020). The challenge of COVID-19 and hematopoietic cell transplantation; EBMT recommendations for management of hematopoietic cell transplant recipients, their donors, and patients undergoing CAR T-cell therapy. Bone Marrow Transplant..

[7] Algwaiz G, Aljurf M, Koh M, Horowitz MM, Ljungman P, Weisdorf D (2020). Real-World Issues and Potential Solutions in Hematopoietic Cell Transplantation during the COVID-19 Pandemic: Perspectives from the Worldwide Network for Blood and Marrow Transplantation and Center for International Blood and Marrow Transplant Research Health Services and International Studies Committee. Biol Blood Marrow Transplant.

[8] Pfefferle S, Reucher S, Nörz D, Lütgehetmann M (2020). Evaluation of a quantitative RT-PCR assay for the detection of the emerging coronavirus SARS-CoV-2 using a high throughput system. Euro Surveill.

[9] Norz D, Frontzek A, Eigner U, Oestereich L, Wichmann D, Kluge S (2020). Pushing beyond specifications: Evaluation of linearity and clinical performance of the cobas 6800/8800 SARS-CoV-2 RT-PCR assay for reliable quantification in blood and other materials outside recommendations. J Clin Virol.

[10] Samek M, Iversen K, Belmar Campos C, Berneking L, Langebrake C, Wolschke C (2020). Monocenter study on epidemiology, outcomes, and risk factors of infections in recipients of 166 allogeneic stem cell transplantations during 1 year. Eur J Haematol.

